# Supplementary material for: Local structure-function relationships in human brain networks across the lifespan
Source: Nat Commun. 2022 Apr 19;13:2053. doi: 10.1038/s41467-022-29770-y (PMC9018911; doi:10.1038/s41467-022-29770-y)
Supplement: Supplementary file 1 — Supplementary Information [file 41467_2022_29770_MOESM1_ESM.pdf]

Supplementary Figures to: Local structure-function relationships in human brain networks across the lifespan

Farnaz Zamani Esfahlani[1], Joshua Faskowitz[1,2], Jonah Slack[1], Bratislav Mišić[3], and Richard F. Betzel[1,2,4,5*]
[1]*Department of Psychological and Brain Sciences, Indiana University, Bloomington, IN 47405*
[2]*Program in Neuroscience, Indiana University, Bloomington, IN 47405*
[3]*McConnell Brain Imaging Centre, Montréal Neurological Institute, McGill University, Montréal, Quebec, Canada* [4]*Cognitive Science Program, Indiana University, Bloomington, IN 47405*
[5]*Network Science Institute, Indiana University, Bloomington, IN 47405*

* rbetzel @ indiana.edu

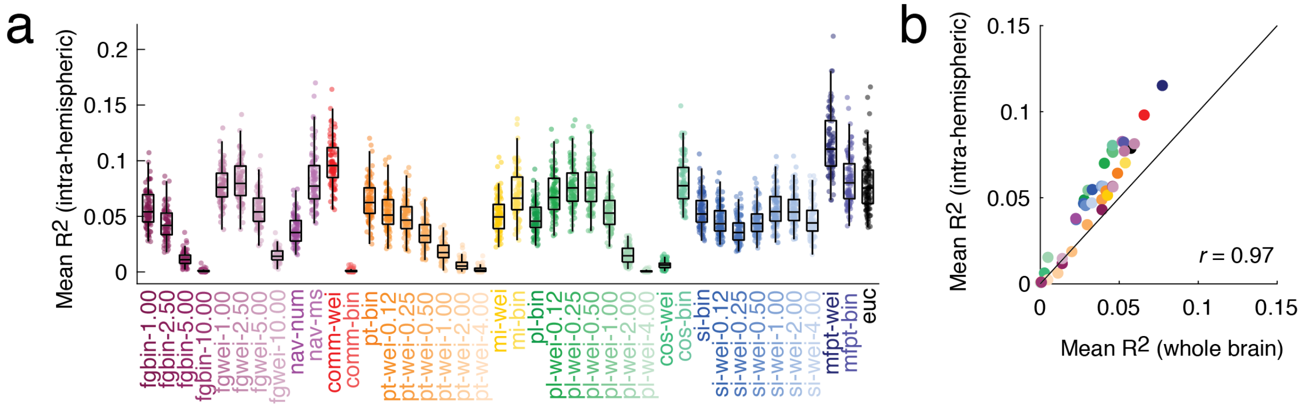

FIG. S1. **Analysis of single hemisphere instead of whole-brain data.** (a) Variance in single-hemisphere FC weights explained by factors. Each point represents a subject ($N_{sub} = 70$ subjects following exclusion for motion and data quality; FC and SC averaged over two scan sessions). To obtain these data, we generated predictors using SC data from the right and left hemispheres separately. Then we used these data to make predictions about FC data from the same hemisphere. Here, we plot the average variance explained over right and left hemispheres. In each boxplot, the "box" denotes interquartile range (IQR), the horizontal bar indicates the median value, and the whiskers include points that are within 1.5 × IQR of upper and lower bounds of the IQR (25th and 7th percentiles). Any points that fall beyond the whiskers are, by convention, considered outliers. (b) Mean R^2 estimated using whole-brain *versus* single-hemisphere data. The black line is an identity line. Note that single-hemisphere data results in improved fits. Source data are provided as a Source Data file.

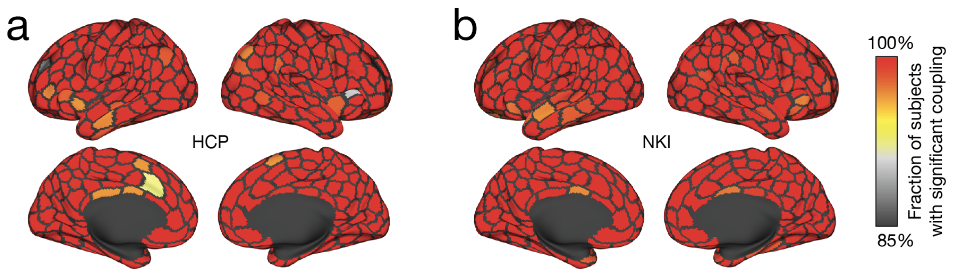

FIG. S2. **Fraction of subjects with significant regional structure-function coupling.** (a) Human Connectome Project dataset. (b) Nathan Kline Institute dataset. For both datasets, the false discovery rate was fixed at $q = 0.05$ and a separate adjusted p-value calculated for each subject. Source data are provided as a Source Data file.

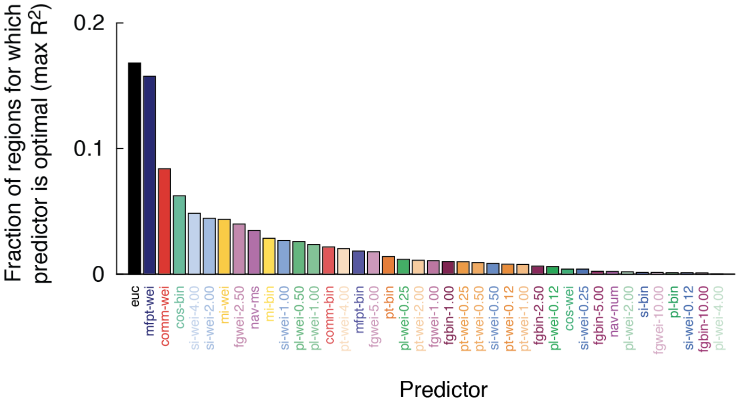

FIG. S3. **Frequency of optimal predictors for modeling local structure-function relationships.** Frequency across subjects and nodes with which each of the 40 predictors best-explained regional patterns of FC. Source data are provided as a Source Data file.

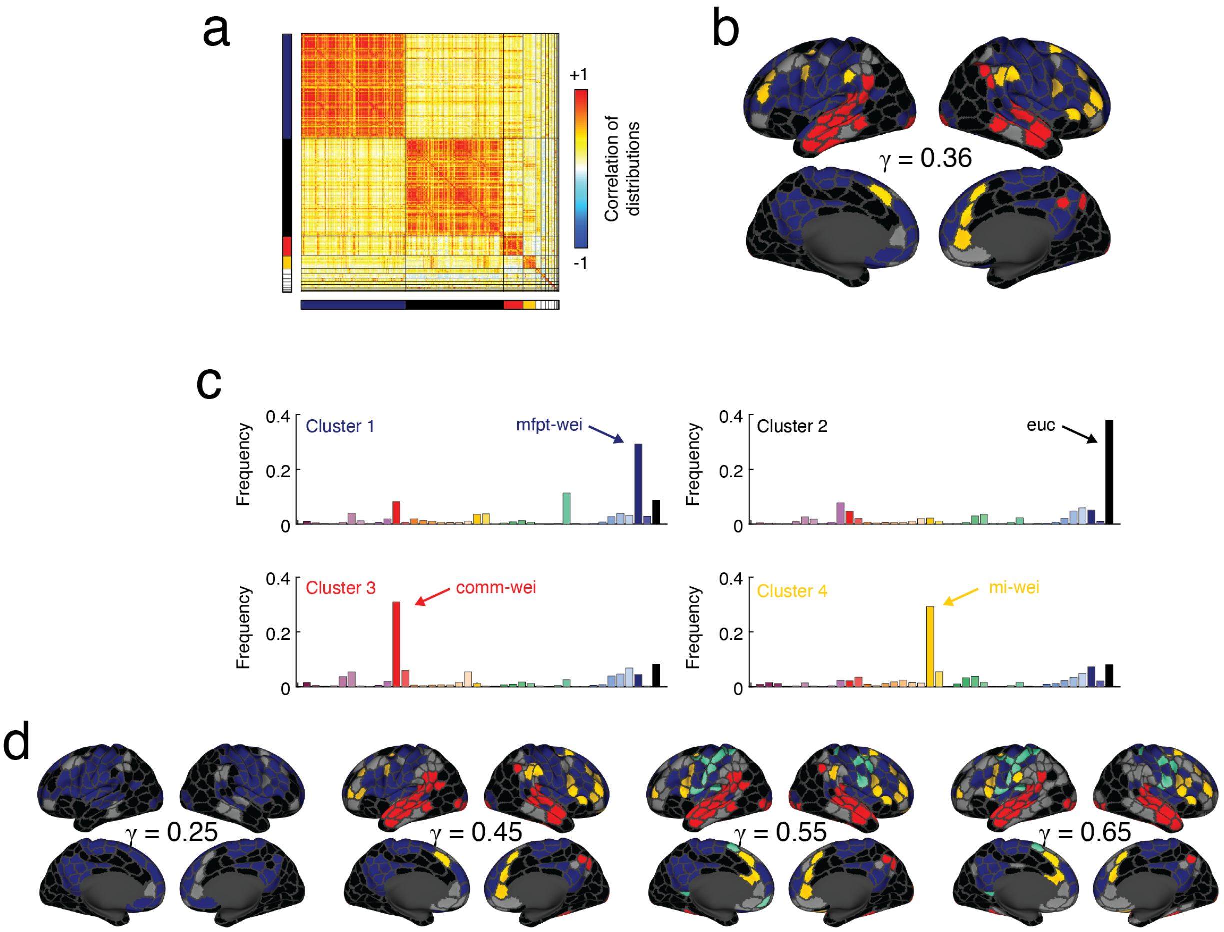

FIG. S4. **Cluster analysis of correlated regional feature vectors.** For each brain region we obtained a 40×1 feature vector whose elements denoted the frequency with which each of the predictors was "optimal" in the HCP dataset. We then computed the region-by-region correlation matrix and used modularity maximization to cluster this matrix and, again, to obtain consensus communities. (a) Correlation matrix ordered by consensus communities. Here we only label the four largest communities based on the structural predictor that best characterizes each community: dark blue (mfpt-wei), black (euc), red (comm-wei), and yellow (mi-wei) . (b) Consensus community assignments projected onto the cortical surface. (c) Mean feature vector for each of the four largest communities. (d) For completeness, we show communities estimated at different resolutions (γ values). Source data are provided as a Source Data file.

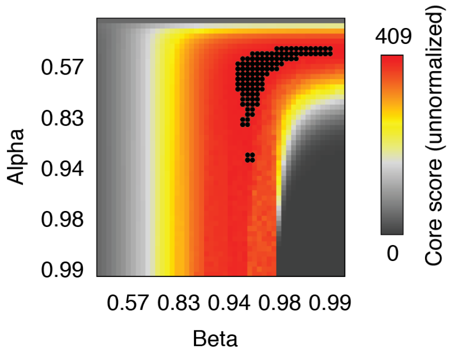

FIG. S5. **Core-periphery analysis.** Core score estimates at every point in parameter space. Black points indicate parameter values (top 5% of core scores) that were included in the core-periphery analysis. Source data are provided as a Source Data file.

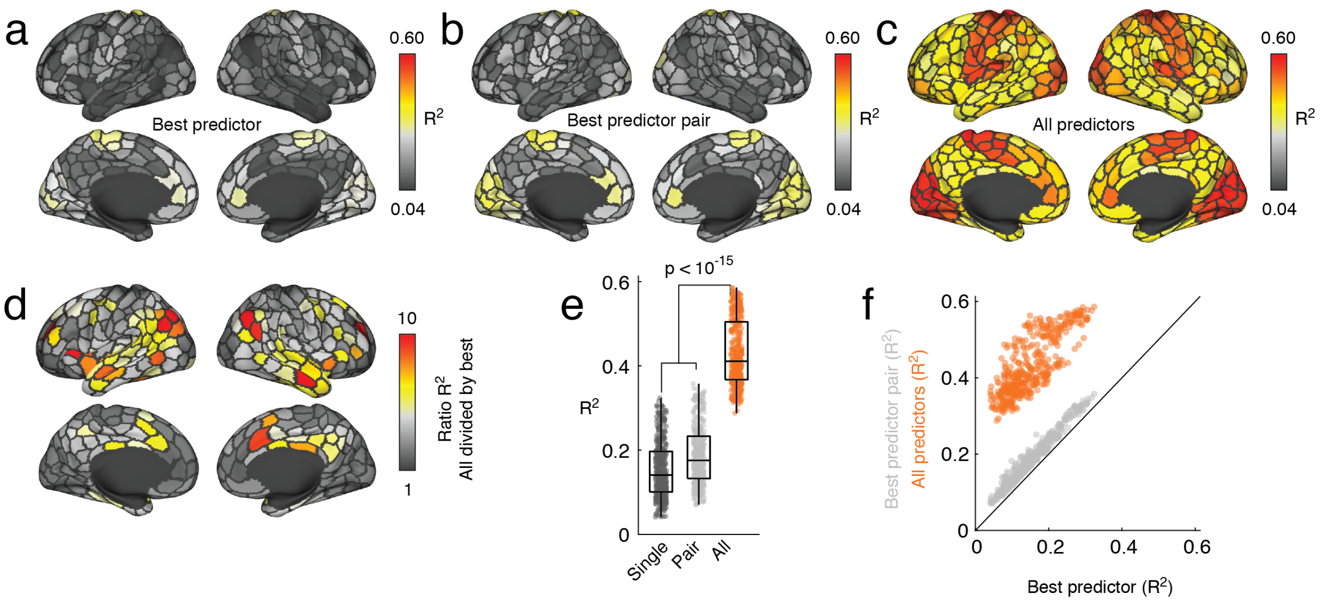

FIG. S6. **Predicting FC using all measures**. In the main text we predicted regional FC using metrics derived from SC matrices. Specifically, we focused on the variance explained by the best individual predictor (a) and the best pair of predictors (b). Here, we repeat this analysis but using *all* predictors to predict regional FC. (c) The variance explained at each region after using all predictors. (d) Ratio of variance explained using the best pair of predictors (panel b) and all predictors (c). Note that the peak increases fall within default mode (interparietal sulcus; posterior cingulate; precuneus) and salience/ventral attention (anterior cingulate). (e) Boxplot of regional variance explained. We compared conditions using paired sample t-tests, two-sided. We corrected for multiple comparisons using the Bonferroni correction. In each boxplot, the "box" denotes interquartile range (IQR), the horizontal bar indicates the median value, and the whiskers include points that are within 1.5 × IQR of upper and lower bounds of the IQR (25th and 7th percentiles). Any points that fall beyond the whiskers are, by convention, considered outliers. (f) Scatterplot of regional variance explained with the single best predictor versus the best pair of predictors (grey) and all predictors (orange). The black line is the identity line. Each point in panels e and f represents a brain region ($N = 400$ parcels defined based on [1]). Source data are provided as a Source Data file.

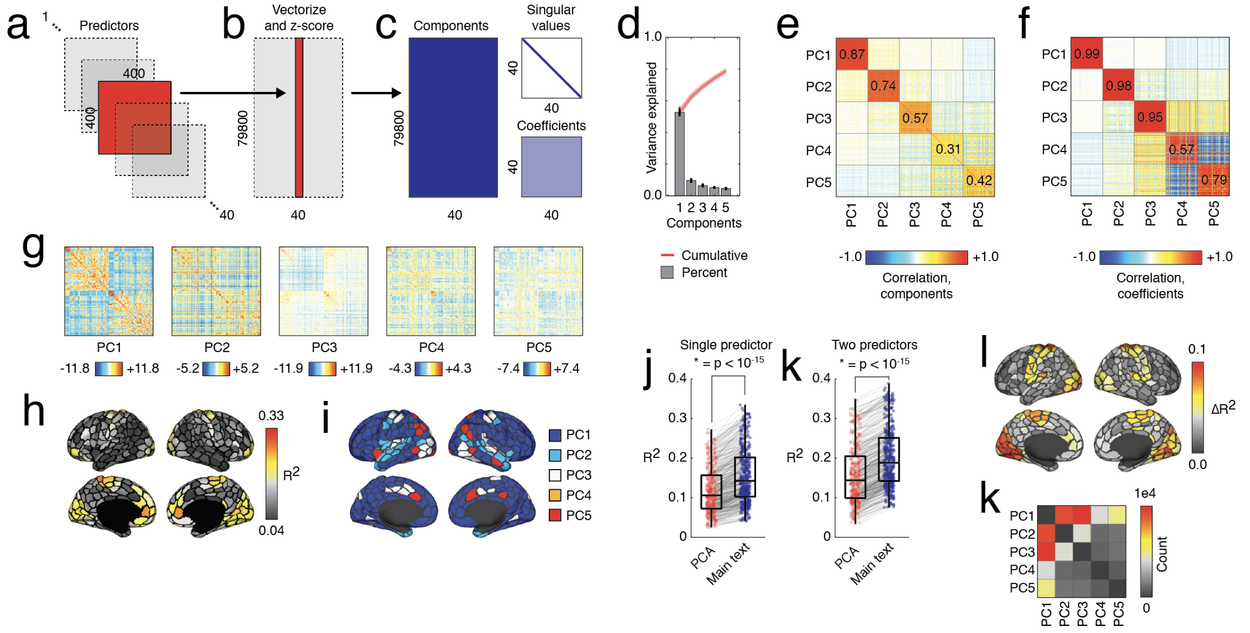

FIG. S7. **Predicting FC using all PCs**. In the main text we predicted regional patterns of FC using a series of predictors and later identified pairs of predictors that maximally improved these predictions. However, because predictors are correlated with one another, interpreting the results of this analysis may be difficult. Here, we use principal component analysis (PCA) to generate an orthonormal basis set from the predictors and use the resulting principal components (PCs) to predict FC. We performed PCA at the subject level using the 40 predictor matrices as input (a). Each matrix was (b) vectorized and z-scored before being decomposed into (c) principal components, coefficients, and singular values. (d) Here, we focus on the top five PCs, which accounted for $>75\%$ of variance across all subjects. We found that the top five PCs explain 52.7 ± 1.3, 9.7 ± 0.5, 6.5 ± 0.4, 5.3 ± 0.2 and 4.6 ± 0.2 percent variance, respectively. In this panel, gray bars correspond to mean variance explained by each PC. The red curves correspond to cumulative variance explained. Despite PCA being performed on individual subjects, the PCs and coefficients were highly correlated across individuals. (e) We found that the similarity of the top five PCs across subjects was 0.87, 0.74, 0.57, 0.30, and 0.42, respectively. This is despite the fact that the dimensionality of each PC was $[79800\times1]$. (f) To similarity of coefficients from the top five PCs was 0.99, 0.98, 0.95, 0.57, and 0.79, respectively. (g) Group-averaged matrix representations of the first five PCs. We used PCs to make predictions about FC, fitting one linear model per region, per PC, and per subject. We identified for each subject the PC that yielded the greatest R^2 and averaged R^2 values across subjects. In panels, h and i we show R^2 values and the optimal PC projected onto cortical surface. We compared R^2 values reported in the main text with those generated using the PCA-based approach. We find that results from the main text outperform those generated using PCs (paired-sample t-test, $p<10^{-15}$). We present this comparison in panel j. As in the main text, we also tested the effect of combining pairs of predictors in the same multilinear model. Again, we found that the two-predictor models reported in the main text outperform the PCA approach (paired sample t-test, $p<10^{-15}$). A boxplot highlighting this comparison is shown in panel k. Points in j and k represent brain regions ($N=400$ parcels in each boxplot defined based on [1]). In both plots we performed two-sided paired sample t-tests. In each boxplot, the "box" denotes interquartile range (IQR), the horizontal bar indicates the median value, and the whiskers include points that are within $1.5\times$ IQR of upper and lower bounds of the IQR (25th and 7th percentiles). Any points that fall beyond the whiskers are, by convention, considered outliers. (l) Increases in R^2 as a results of including an additional PC as a predictor. Interestingly, the combinations of predictors that lead to the greatest improvement tended to involve PC1 pairing with other components. (k) A matrix plot of how frequently pairs of predictors were identified as the optimal model. Source data are provided as a Source Data file.

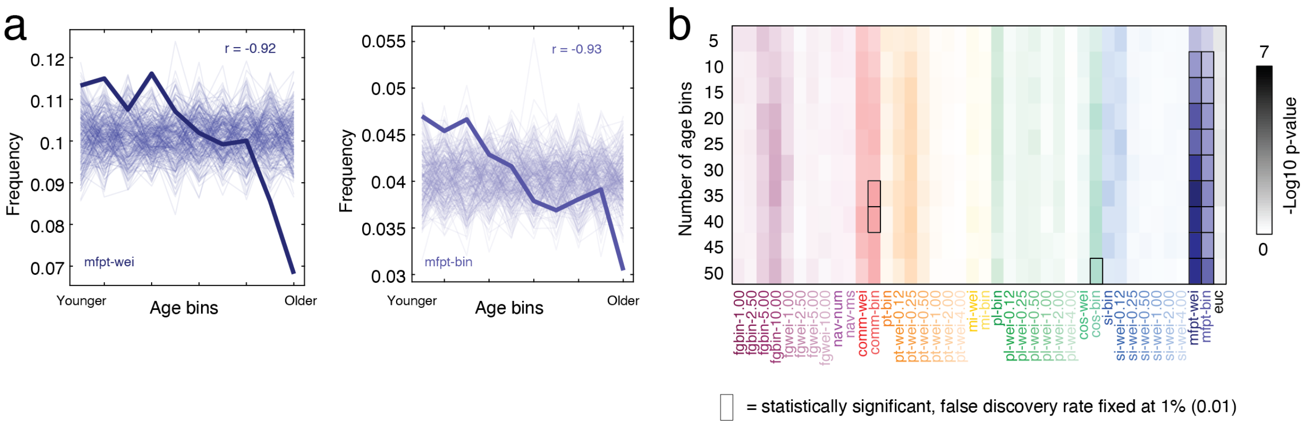

FIG. S8. **Effect of bin size on the correlation of predictor frequency with age**. In the main text we reported a correlation between the frequency with which given predictors are optimal for a given region and age. In that analysis, each data point corresponded to an individual. Here, we partitioned subjects into age bins of varying size, calculated the mean age and frequency of each bin, and computed the correlation between these bin-level estimates. Here, we report results from this analysis. (a) With a bin size of 10, we find that only the weighted and binary mean first passage times were correlated with age. (b) In general, we show that these correlations persist across a broad range of age bins (from 5 to 50 in increments of 5). We also find that other measures are correlated at different bin sizes, but not consistently. In b, cell correspond to pairs of predictors and bin sizes. The elements in each cell are p-values associated with correlations between predictor frequency and bin size. We corrected for multiple comparisons by adjusting the critical p-value to achieve a false positive rate of $q = 0.05$ (5%). Source data are provided as a Source Data file.

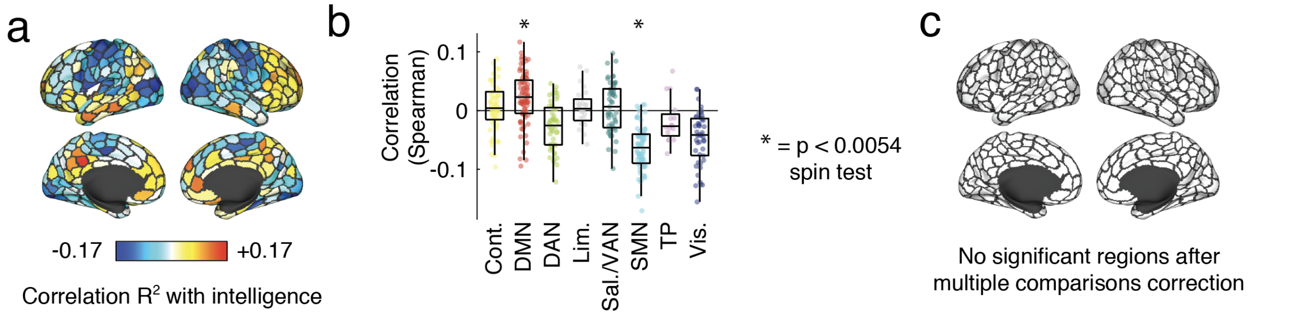

FIG. S9. **Structure-function relationships are linked to intelligence.** We calculated the regional correlation of the structure-function coupling (R^2) with four measures of intelligence: (1) Wechsler Individual Achievement Test Composite score, (2-4) Wechsler Abbreviated Scale of Intelligence full scale IQ, verbal comprehension, and perceptual reasoning index. These scores were highly correlated ($r = 0.81 \pm 0.10$) and we report their mean correlation with R^2. (a) Spatial distribution of intelligence-R^2 associations projected onto cerebral cortex. (b) Correlation coefficients grouped by brain system. We compared the mean correlation of systems with a null distribution generated using a spin test (1000 permutations) and found that positive correlations were overly expressed in the default mode while negative correlations were overly expressed in the somatomotor network. In b, * indicates that the mean correlation of parcels in a given system was significantly greater than the null distribution generated under the spin test. We controlled for multiple comparisons by adjusted the critical p-value to coincide with a false discovery rate of $q = 0.05$ (5%). The exact p-values for the default mode and somatomotor networks were $p = 5.75 \times 10^{-5}$ and $p = 2.5 \times 10^{-4}$, respectively. In each boxplot, the "box" denotes interquartile range (IQR), the horizontal bar indicates the median value, and the whiskers include points that are within 1.5 × IQR of upper and lower bounds of the IQR (25th and 7th percentiles). Any points that fall beyond the whiskers are, by convention, considered outliers. (c) Thresholded correlation map. Note that no regions pass statistical tests following multiple comparison corrections. Source data are provided as a Source Data file.

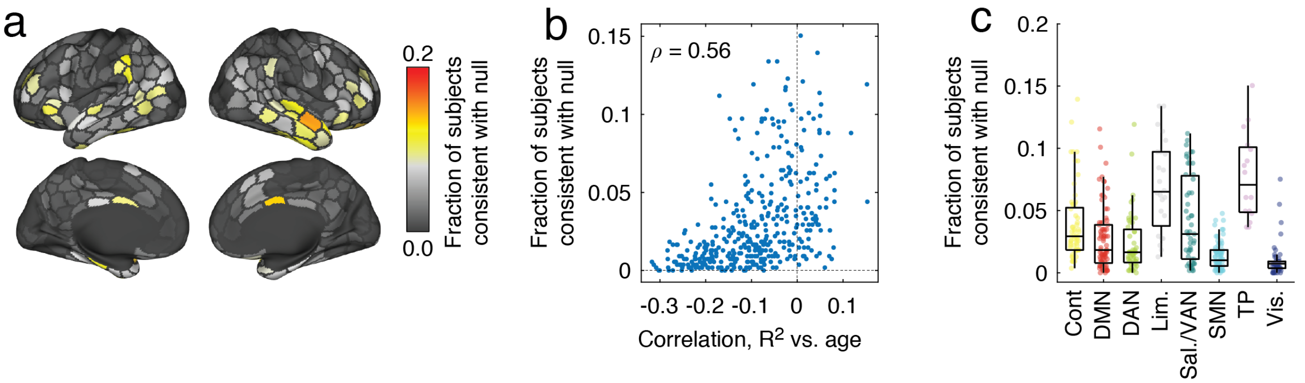

FIG. S10. **Floor effect in NKI dataset.** In the main text we reported a correlation between regional R^2 and age using the NKI dataset. Here we assess the possibility that the spatial pattern of these correlations may be driven, in part, by a floor effect. To test for this effect, we use a permutation-based null model. Regions whose R^2 values cannot be statistically distinguished from the null distribution are especially susceptible to a floor effect. In more detail, this procedure entailed the following. For each subject, we predicted their regional FC patterns using predictors derived from a randomized SC matrix (the order of its rows and columns were permuted). We then calculated and retained, for each region, its maximum R^2 across any of the predictors. We repeated this procedure 100 times for each subject, generating subject-specific null distributions. We compared the observed R^2 values with this null distribution (z-score) and identified regions whose z-statistic could not be distinguished statistically from the null distribution($p \geq 0.05$). We counted how frequently across subjects each of the 400 regions was part of this group, considering them to have already reached their floor R^2. We find that, on average, most regions exhibit R^2 values that are statistically greater than their theoretical floor ($96.8 \pm 1.5\%$). The specificity of regions that are approaching their R^2 floor is also poor; the region with the greatest frequency is consistent with the null distribution in 14.7% of subjects. Interestingly, and as the reviewer anticipated, the whole-brain pattern is similar to the correlation pattern reported in the main text ($\rho = 0.56$; $p < 10^{-15}$), opening up the possibility that some of the age-related differences may be attributable to a floor effect. (a) Fraction of subjects in which a region's R^2 is not distinguishable from the null distribution. We show these values projected onto the cortical surface. (b) Values from panel a with the regional R^2 *versus* age correlation coefficients. (c) Values from panel a grouped by system. Each point represents a brain region ($N = 400$ parcels defined based on [1]). In each boxplot, the "box" denotes interquartile range (IQR), the horizontal bar indicates the median value, and the whiskers include points that are within $1.5 \times$ IQR of upper and lower bounds of the IQR (25th and 7th percentiles). Any points that fall beyond the whiskers are, by convention, considered outliers. Source data are provided as a Source Data file.

REFERENCES

[1] Alexander Schaefer, Ru Kong, Evan M Gordon, Timothy O Laumann, Xi-Nian Zuo, Avram J Holmes, Simon B Eickhoff, and BT Thomas Yeo, "Local-global parcellation of the human cerebral cortex from intrinsic functional connectivity mri," Cerebral cortex **28**, 3095–3114 (2018).
