# Supplementary material for: Local structure-function relationships in human brain networks across the lifespan
Source: Nat Commun. 2022 Apr 19;13:2053. doi: 10.1038/s41467-022-29770-y (PMC9018911; doi:10.1038/s41467-022-29770-y)
Supplement: Supplementary file 3 — Description of Additional Supplementary Information [file 41467_2022_29770_MOESM3_ESM.pdf]

The supplementary information contains 10 figures.

Figure S1. Analysis of single hemisphere instead of whole-brain data

Figure S2. Fraction of subjects with significant regional structure-function coupling.

Figure S3. Frequency of optimal predictors for modeling local structure-function relationships

Figure S4. Cluster analysis of correlated regional feature vectors

Figure S5. Core-periphery analysis

Figure S6. Predicting FC using all measures

Figure S7. Predicting FC using all PCs

Figure S8. Effect of bin size on the correlation of predictor frequency with age

Figure S9. Structure-function relationships are linked to intelligence

Figure S10. Floor effect in NKI dataset.
