# Supplementary material for: Local structure-function relationships in human brain networks across the lifespan
Source: Nat Commun. 2022 Apr 19;13:2053. doi: 10.1038/s41467-022-29770-y (PMC9018911; doi:10.1038/s41467-022-29770-y)
Supplement: Supplementary file 4 — Supplementary figure 1 [file 41467_2022_29770_MOESM4_ESM.docx]

How regional anatomy shapes function is not well understood. Here, the authors evaluate the performance of 40 communication models in predicting FC. They find regional heterogeneity in terms of fit and optimal model. They also find that regional coupling varies over the human lifespan.
