# Supplementary figures and images for: Local structure-function relationships in human brain networks across the lifespan
Source: Nat Commun. 2022 Apr 19;13:2053. doi: 10.1038/s41467-022-29770-y (PMC9018911; doi:10.1038/s41467-022-29770-y)

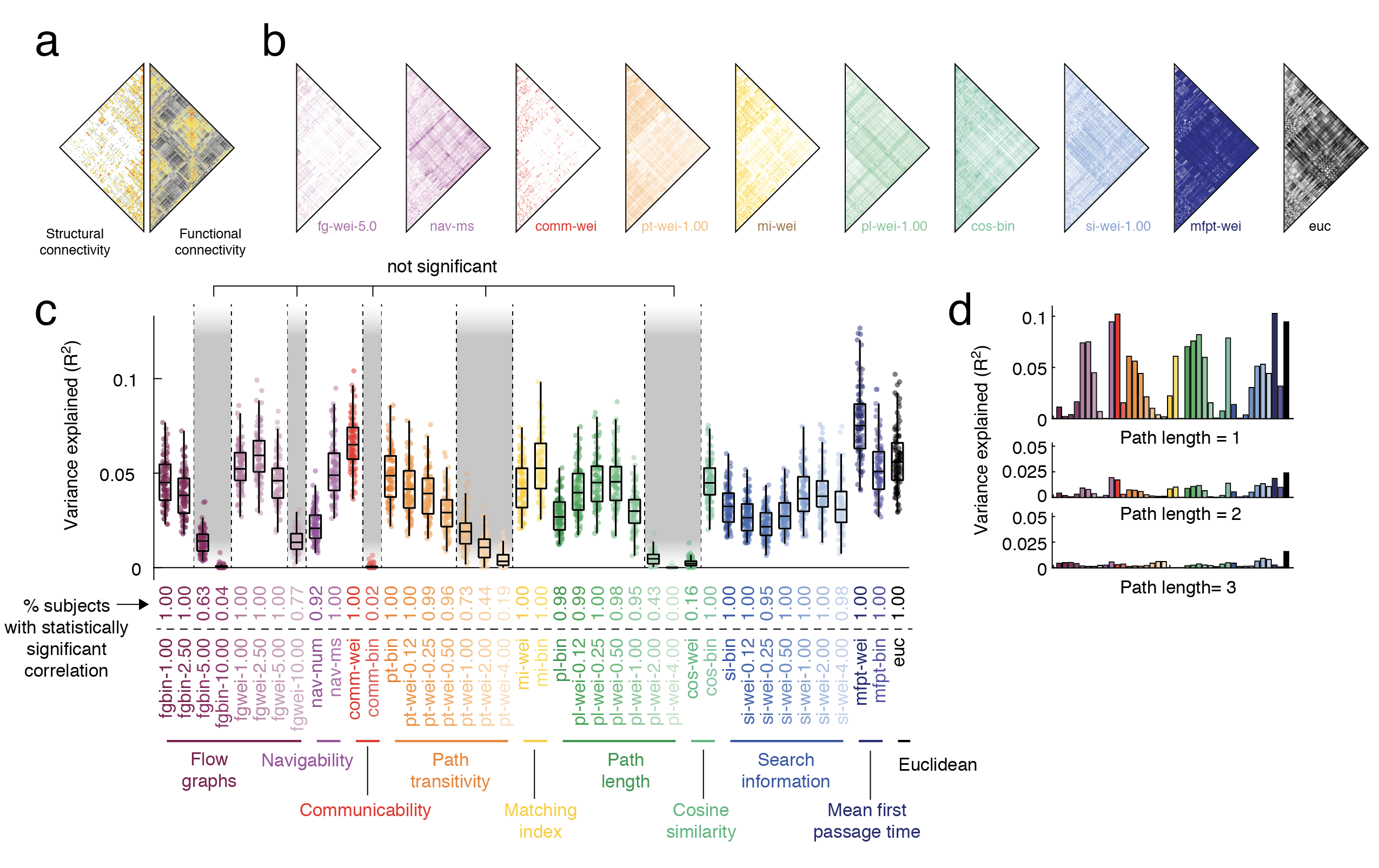

Supplement: Supplementary file 5 — Supplementary figure 2 [file 41467_2022_29770_MOESM5_ESM.png]

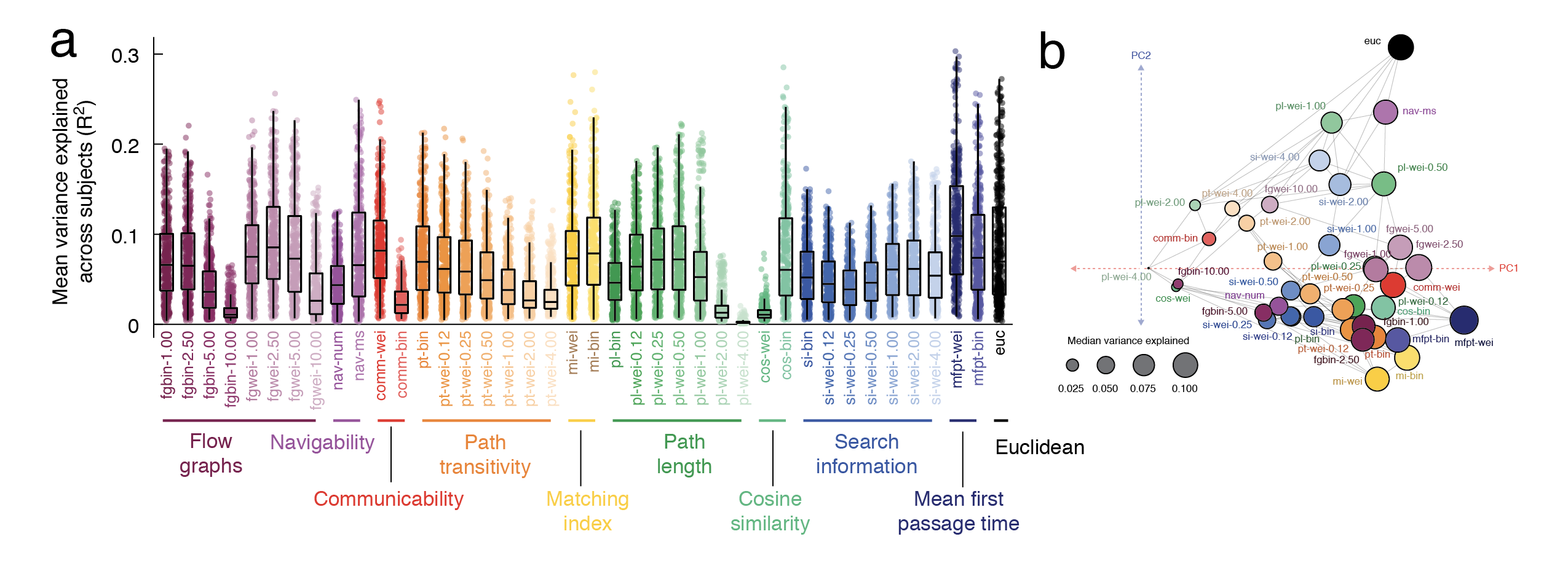

Supplement: Supplementary file 6 — Supplementary figure 3 [file 41467_2022_29770_MOESM6_ESM.png]
