# Supplementary material for: Local structure-function relationships in human brain networks across the lifespan
Source: Nat Commun. 2022 Apr 19;13:2053. doi: 10.1038/s41467-022-29770-y (PMC9018911; doi:10.1038/s41467-022-29770-y)
Supplement: Supplementary file 7 — Reporting Summary [file 41467_2022_29770_MOESM7_ESM.pdf]

nature research

Corresponding author(s): Richard Betzel

Last updated by author(s): Feb 3, 2022

Reporting Summary

Nature Research wishes to improve the reproducibility of the work that we publish. This form provides structure for consistency and transparency in reporting. For further information on Nature Research policies, see our Editorial Policies and the Editorial Policy Checklist.

Statistics

For all statistical analyses, confirm that the following items are present in the figure legend, table legend, main text, or Methods section.

n/a	Confirmed	
☐	☒	The exact sample size (n) for each experimental group/condition, given as a discrete number and unit of measurement
☐	☒	A statement on whether measurements were taken from distinct samples or whether the same sample was measured repeatedly
☐	☒	The statistical test(s) used AND whether they are one- or two-sided<br>*Only common tests should be described solely by name; describe more complex techniques in the Methods section.*
☐	☒	A description of all covariates tested
☐	☒	A description of any assumptions or corrections, such as tests of normality and adjustment for multiple comparisons
☐	☒	A full description of the statistical parameters including central tendency (e.g. means) or other basic estimates (e.g. regression coefficient) AND variation (e.g. standard deviation) or associated estimates of uncertainty (e.g. confidence intervals)
☐	☒	For null hypothesis testing, the test statistic (e.g. F, t, r) with confidence intervals, effect sizes, degrees of freedom and P value noted<br>*Give P values as exact values whenever suitable.*
☒	☐	For Bayesian analysis, information on the choice of priors and Markov chain Monte Carlo settings
☒	☐	For hierarchical and complex designs, identification of the appropriate level for tests and full reporting of outcomes
☐	☒	Estimates of effect sizes (e.g. Cohen's d, Pearson's r), indicating how they were calculated

Our web collection on statistics for biologists contains articles on many of the points above.

Software and code

Policy information about availability of computer code

Data collection	The authors of this study did not collect any of the analyzed data; therefore the authors did not use software for data collection.
Data analysis	Human Connectome Project data are provided already minimally preprocessed at the ConnectomeDB (https:/db.humanconnectome.org/app/template/Login.vm). Subject specific parcellations were fit with FreeSurfer 6.0.1 using code available here: https://github.com/faskowit/multiAtlasTT and data available here: https://figshare.com/articles/multiAtlasTT_data_hcptrained/7552853. fMRI data were nuisance regressed with code available here: https://github.com/faskowit/app-fmri-2-mat which uses Nilearn's signal.clean, from Nilearn 0.5.0.<br><br>Custom code used to implement communication policies and to fit models to data are available here: https://github.com/brain-networks/local_scfc.

For manuscripts utilizing custom algorithms or software that are central to the research but not yet described in published literature, software must be made available to editors and reviewers. We strongly encourage code deposition in a community repository (e.g. GitHub). See the Nature Research guidelines for submitting code & software for further information.

Data

Policy information about availability of data

All manuscripts must include a data availability statement. This statement should provide the following information, where applicable:

- Accession codes, unique identifiers, or web links for publicly available datasets
- A list of figures that have associated raw data
- A description of any restrictions on data availability

All imaging data come from publicly-available, open-access repositories. Human Connectome Project data can be accessed at: https://db.humanconnectome.org/

app/template/Login.vm, after signing a data use agreement. Then Enhanced Nathan Kline Institute - Rockland Sample can be accessed at: http://fcon_1000.projects.nitrc.org/indi/enhanced/neurodata.html.

Field-specific reporting

Please select the one below that is the best fit for your research. If you are not sure, read the appropriate sections before making your selection.

☒ Life sciences ☐ Behavioural & social sciences ☐ Ecological, evolutionary & environmental sciences

For a reference copy of the document with all sections, see nature.com/documents/nr-reporting-summary-flat.pdf

Life sciences study design

All studies must disclose on these points even when the disclosure is negative.

Sample size	Two human neuroimaging datasets were used in this study: Human Connectome Project (HCP) and Enhanced Nathan Kline Institute - Rockland Sample (NKI). For HCP, we used a publicly available list of 100 unrelated subjects. For NKI, the collection of a large-scale community sample of neuroimaging is ongoing and data for this project were downloaded from the NKI Amazon S3 bucket in December 2016. No statistical methods were used to predetermine sample sizes, but our sample sizes are similar to those reported in previous publications and represent either all usable and complete data at the time of download (NKI) or a subset preselected by the study coordinators (HCP).
Data exclusions	For our neuroimaging data, data exclusions were based on data quality, to filter out scans with excessive motion or image artifact, and based on data completeness, to ensure that each subject that the appropriate meta-data, and both structural and functional scans. For HCP, subjects were considered for data exclusion based on the mean and mean absolute deviation of the relative root-mean-square motion across either four resting-state MRI scans (file: Movement_RelativeRMS.txt) or one diffusion MRI scan (file: eddy_unwarped_images.eddy_movement_rms), resulting in four summary motion measures. If a subject exceeded 1.5 times the interquartile range (in the adverse direction) of the measurement distribution in two or more of these measures, the subject was excluded. These exclusion criteria were established before the current study. Four subjects were excluded based on these criteria. One subject was excluded for software error during diffusion MRI processing. For NKI, subjects were considered for data exclusion based on having a complete set of T1w, resting state, diffusion images, and meta-data, as well as the quality of the aforementioned images. The ENIGMA QC FreeSurfer tools (http://enigma.ini.usc.edu/protocols/imaging-protocols/), MRIQC (https://mriqc.readthedocs.io/en/stable/), eddy_qc (https://fsl.fmrib.ox.ac.uk/fsl/fslwiki/eddyqc/UsersGuide), and QAscripts (https://www.med.upenn.edu/cmroi/qascripts.html) were used to derive image quality metrics to assess data quality of the T1w, resting state, and diffusion images. Details of exclusion criteria can be found in the manuscript section "Quality Control". Of the 567 subjects who passed image quality control, only 542 subjects had age meta-data available.
Replication	We analyze two independently acquired datasets and report strong correlations between regional coupling patterns. We also separately analyzed whole-brain and single-hemisphere data to test whether effects might be driven by inability to reconstruct cross callosal fibers. We found a strong correspondence.
Randomization	Subjects were not partitioned into groups. Data from each cohort (HCP, NKI) were analyzed separately. This choice was made so as to not mix data across MRI machine and MRI acquisition parameters.
Blinding	Data analysis was not performed blind to the conditions of the experiments. Blinding was not relevant because subjects were not evaluated based on group membership and blinding was not applicable to the whole-group analyses reported in this study.

Reporting for specific materials, systems and methods

We require information from authors about some types of materials, experimental systems and methods used in many studies. Here, indicate whether each material, system or method listed is relevant to your study. If you are not sure if a list item applies to your research, read the appropriate section before selecting a response.

Materials & experimental systems

n/a	Involved in the study	
☒	☐	Antibodies
☒	☐	Eukaryotic cell lines
☒	☐	Palaeontology and archaeology
☒	☐	Animals and other organisms
☐	☒	Human research participants
☒	☐	Clinical data
☒	☐	Dual use research of concern

Methods

n/a	Involved in the study	
☒	☐	ChIP-seq
☒	☐	Flow cytometry
☐	☒	MRI-based neuroimaging

Human research participants

Policy information about studies involving human research participants

Population characteristics	The Human Connectome Project (HCP) aimed to collect healthy adult twins, ages 22-35 years old (Van Essen, 2012). The

Population characteristics	definition of "healthy" was broad, in order to collect a sample representative of the United States population in terms of behavior, ethnic, and socioeconomic diversity. In this study, a provided subset of subjects called the "Unrelated 100" was used (56% female). The Enhanced Nathan Kline Institute - Rockland Sample (NKI) is a prospective data collection project (which began in March 2012) aiming to collect a large scale (N>1000) community-ascertained lifespan sample (ages 6-85 years old; 56% female). The sample was calculated to be 0.32% of the population of Rockland County, New York. The study coordinators aimed to collect a sample with representative age, ethnicity, and socioeconomic status of Rockland County, New York. Details of of these sample objectives can be found in Nooner et. al (2012).
Recruitment	HCP subjects were recruited from the Missouri Department of Health and Senior Services Bureau of Vital Records. NKI subjects were recruited based on zip code (e.g. advertisement flyer mailings, posting of recruitment materials in local shops and meeting places). The authors of the present study did not collect any of the primary imaging data nor did they conduct recruitment of subjects. Therefore, the authors are not knowledgeable about possible self-section biases or other biases associated with the data collection of these two cohorts. We would estimate that potential biases related to recruitment would not likely impact the main results of the study.
Ethics oversight	HCP was approved by the Washington University Institutional Review Board. NKI was approved by the Institutional review boards at Nathan Kline Institute and at Montclair State University.

Note that full information on the approval of the study protocol must also be provided in the manuscript.

Magnetic resonance imaging

Experimental design

Design type	In this study, we used T1-weighted, resting state (fixation cross, eyes open), and diffusion weighted MRI scans
Design specifications	No blocks were used in this study for these image acquisitions.
Behavioral performance measures	Behavioral measures were used were not used for the main findings of this study. In Supplementary Figure 6, we use the Wechsler Individual Achievement Test Composite score, the Wechsler Abbreviated Scale of Intelligence full scale IQ, verbal comprehension, and perceptual reasoning index scores to relate to structure-function relationships.

Acquisition

Imaging type(s)	structural, functional, diffusion
Field strength	3T
Sequence & imaging parameters	For HCP, a comprehensive description of the imaging parameters and image prepocessing can be found in Glasser et al. 2013. Images were collected on a 3T Siemens Connectome Skyra with a 32-channel head coil. Subjects underwent two T1-weighted structural scans, which were averaged for each subject (TR = 2400 ms, TE = 2.14 ms, flip angle = 8◦ , 0.7 mm isotropic voxel resolution). Subjects underwent four resting state fMRI scans over a two-day span. The fMRI data was acquired with a gradient-echo planar imaging sequence (TR = 720 ms, TE = 33.1 ms, flip angle = 52◦ , 2 mm isotropic voxel resolution, multiband factor = 8). Each resting state run duration was 14:33 min, with eyes open and instructions to fixate on a cross. Finally, subjects underwent two diffusion MRI scans, which were acquired with a spin-echo planar imaging sequence (TR = 5520 ms, TE = 89.5 ms, flip angle = 78◦ , 1.25 mm isotropic voxel resolution, b-vales = 1000, 2000, 3000 s/mm2 , 90 diffusion weighed volumes for each shell, 18 b = 0 volumes). These two scans were taken with opposite phase encoding directions and averaged.<br>For NKI, comprehensive description of the imaging parameters can be found on NKI's website: http://fcon_1000.projects.nitrc.org/indi/enhanced/mri_protocol.html. Briefly, images were collected on a Siemens Magneton Trio with a 12-channel head coil. Subjects underwent one T1-weighted structural scan (TR = 1900 ms, TE = 2.52 ms, flip angle = 9◦ , 1 mm isotropic voxel resolution). Subjects underwent three differently parameterized resting state scans, but only one acquisition is used in the present study. The fMRI data was acquired with a gradient-echo planar imaging sequence (TR = 645 ms, TE = 30 ms, flip angle = 60◦ , 3 mm isotropic voxel resolution, multiband factor = 4). This resting state run lasted approximately 9:41 seconds, with eyes open and instructions to fixate on a cross. Subjects underwent one diffusion MRI scan (TR = 2400 ms, TE = 85 ms, flip angle = 90◦ , 2 mm isotropic voxel resolution, 128 diffusion weighted volumes, b-value = 1500 s/mm2 , 9 b = 0 volumes).
Area of acquisition	whole brain

Diffusion MRI ☒ Used ☐ Not used

Parameters	HCP: 3 shells (bvals: 1000, 2000, 3000), 90 directions per shell, 18 unweighted volumes; NKI: 1 shell (bval: 1500), 128 directions, 9 unweighted volumes

Preprocessing

Preprocessing software	Functional, anatomical, and diffusion images in the HCP were minimally processed with the HCP pipelines and downloaded after the 1200 subject data release (for documentation, see: https://www.humanconnectome.org/study/hcp-young-adult/document/1200-subjects-data-release). The HCP pipelines utilize FSL (version 5.0.6), FreeSurfer (version 5.3HCP), Connectome Workbench (versio 1.1.1), and custom MATLAB (pipelines available here: https://github.com/Washington-University/HCPpipelines; and described in Glasser et al. 2013). To perform white matter model fitting and streamline

tractography we used Dipy 1.1.
Functional and anatomical images in the NKI dataset were preprocessed using fMRIPrep 1.1.8, which is based on Nipype. Internal operations of fMRIPrep use Nilearn 0.5.0, ANTs 2.1.0, FreeSurfer 6.0.1, FSL 5.0.9, and AFNI v16.2.07. For more details about the pipeline, see the section corresponding to workows in fMRIPrep's documentation, for version 1.1.8 (https://fmriprep.org/en/stable/citing.html). Diffusion images were preprocessed following the DESIGNER protocol (Ades-Aron et al., 2018) which uses MRTrix 3.0 functions. To perform white matter model fitting and streamline tractography we used Dipy 0.16.0.

Normalization	Within the fMRIPrep workflow, ANTs is used to align functional images to the MNI Asymmetrical template version 2009c. For HCP, FSL FNIRT is used to align functional images in the FSL MNI template and furthermore, multi-modal registration is used to align surface functional data to the fs_LR surface space. For rendering volumetric parcellations in the T1w anatomical space for both HCP and NKI, FreeSurfer's (version 6.0.1) mris_ca_label function was used in conjunction pre-trained Gaussian classifier surface atlases (found here: https://figshare.com/articles/dataset/multiAtlasTT_data_hcptrained/7552853).
Normalization template	For HCP, fMRI data was analyzed after linear alignment (AC-PC) to the FSL MNI template. For NKI, fMRI data was analyzed in each subject's T1w space.
Noise and artifact removal	For functional data of the HCP and NKI, we employed a 36-parameter nuisance regression strategy described Satterthwaite et. al (2013) and shown to be a relatively effective strategy (with and without spike regression) in Parkes et. al (2018). For diffusion images of the HCP, data is provided preprocessed. For diffusion images of the NKI, FSL's eddy was run with the '--repol' option.
Volume censoring	We used spike regression to perform volume censoring. For NKI, spike regressor was added for each fMRI frame exceeding 0.5 mm framewise displacement. For HCP, spike regression was not applied.<br><br>In the supplement we reanalyzed HCP and NKI data employing a more rigorous motion threshold. Specifically, following preprocessing (which was left unchanged), we:<br>1. censored any frames with relative motion > 0.15,<br>2. censored frames that were within 2 or fewer frames of any frame dropped in step 1, and<br>3. retained only contiguous sequences of frames of length 5.<br>4. In addition, we completely excluded any scans that contained fewer than 50% of usable frames following steps 1-3. Any subject dropped a scan based on this criterion was also removed from all subsequent analyses.

Statistical modeling & inference

Model type and settings	Mass univariate
Effect(s) tested	Statistical association of regional structure-function coupling with biological age. Assessed using multi-linear models.

Specify type of analysis: ☒ Whole brain ☐ ROI-based ☐ Both

Statistic type for inference (See Eklund et al. 2016)	Correlation coefficient
Correction	FDR correction

Models & analysis

n/a	Involved in the study
☐	☒ Functional and/or effective connectivity
☐	☒ Graph analysis
☒	☐ Multivariate modeling or predictive analysis

Functional and/or effective connectivity	We constructed functional and structural connectivity for every subject in two datasets and using "communication dynamics" models to link the two modalities to one another.
Graph analysis	Weighted and sparse structural connectivity matrices. Fully-weighted and signed functional connectivity matrices (correlation).
